# Barriers and Facilitators to Long-Term Services & Supports for Older Adults Living Alone With Cognitive Impairment

**DOI:** 10.1093/geroni/igaf122.3718

**Published:** 2025-12-31

**Authors:** Elena Portacolone, Robyn Stone, Matthew Growdon, Thi Tran, Daisy Feddoes, Andrew Cohen

**Affiliations:** University of California, San Francisco, San Francisco, California, United States; LeadingAge, Washington, District of Columbia, United States; University of California, San Francisco, San Francisco, California, United States; University of California, San Francisco, San Francisco, California, United States; University of California, San Francisco, San Francisco, California, United States; Yale School of Medicine, New Haven, Connecticut, United States

## Abstract

**Background:**

More than 4 million older Americans with cognitive impairment (CI) live alone. These individuals often face unique challenges due to limited or non-existent support from caregivers. Identifying the barriers and facilitators to accessing long-term services and supports (LTSS) is critical to preventing the adverse outcomes to which this population is particularly vulnerable, including self-neglect, untreated medical conditions, medication mismanagement, falls, and fires.

**Methods:**

This qualitative study, conducted between May 2016-February 2024, included adults aged 55+ from diverse racial and ethnic backgrounds who were living alone with CI in California, Louisiana, and Michigan, as well as members of their social circle. Factors influencing LTSS access were identified via in-person, semi-structured interviews conducted in English, Spanish, Cantonese, or Mandarin. Transcripts were analyzed with combined inductive and deductive content analysis drawing from the micro-meso-macro framework.

**Results:**

A total of 119 older adults living alone with cognitive impairment (88 [71.5%] women; median age, 78 years [range, 57-103]) and 56 members of their social circle (45 [80.3%] women; median age, 45 years [range, 39-89]) were interviewed multiple times for a total of 558 interviews. Barriers to accessing LTSS included: healthcare providers being unhelpful, distrustful, dismissive, and unaffordable, with rigid protocols and long waiting periods; cognitive impairment itself in the context of living alone; limited resources.

**Facilitators included:**

having chronic medical conditions; “hunting” for services; information from attentive healthcare providers; support with transportation.

**Conclusions:**

Findings underscore the importance of ensuring that critical LTSS are tailored around the needs of older adults living alone with CI.

